# Surgical outcomes of cutaneous squamous cell carcinoma in the head and neck: analysis of resection margins, recurrences, and metastasis

**DOI:** 10.3389/fonc.2026.1766397

**Published:** 2026-02-13

**Authors:** Felix Deffner, Anna Charlotta Schlieper, Givi Magradze, Christoph Becker, Naglaa Mansour, Andreas Knopf, Susanne M. Moldenhauer, Kia Melzer, Manuel Christoph Ketterer

**Affiliations:** Department of Otorhinolaryngology, Medical Center – University of Freiburg, Faculty of Medicine, University of Freiburg, Freiburg im Breisgau, Germany

**Keywords:** cutaneous squamous cell carcinoma (cSCC), head and neck, metastasis, recurrence, resection margin, surgical outcome

## Abstract

**Introduction:**

Cutaneous squamous cell carcinoma (cSCC) represents the second most common skin cancer and poses a significant public health challenge. Given the rising incidence of skin cancer, it is essential to address surgical management strategies and to identify factors associated with clear resection margins, recurrence, and metastasis. This study aims to analyze the frequency of tumor-free resection margins, local recurrence, and metastasis, as well as the underlying risk factors. The central research question is: Which clinical and histopathological characteristics are significantly associated with an increased risk of incomplete resection, recurrence, and metastasis?

**Materials and methods:**

A retrospective analysis was conducted on data from 176 patients diagnosed with cSCC between 2019 and 2024. Patient data were evaluated based on clinical records, histopathological findings (including tumor thickness and depth, histological subtype, infiltration pattern, and surgical margin status), and follow-up assessments, which included routine ultrasound examinations.

**Results:**

Tumor diameter was the only independent predictor of achieving tumor-free resection margins (R0 status). Local recurrence occurred in 11.2% of patients and was significantly associated with tumor depth, whereas tumor diameter showed no correlation with recurrence-free survival. Suspicious lymph nodes on preoperative ultrasonography were detected in 3% of patients in the neck and 5% in the parotid gland. Four patients (2.2%) with suspicious findings underwent neck dissection and/or parotidectomy; synchronous metastases were confirmed in three cases (1.7%), all in cervical nodes. Further five patients (2.8%) developed metachronous lymph node metastases and were treated with surgery and adjuvant radiotherapy.

**Discussion:**

Tumor depth was identified as the main determinant for achieving complete tumor resection, underscoring its prognostic relevance in cSCC. The low incidence of synchronous and metachronous lymph node metastases highlights the favorable local control achievable with adequate surgical margins and regular follow-up. The study further emphasizes the importance of early detection and structured surveillance, including regular head and neck ultrasonography, for the timely identification of metastatic disease and optimized patient management.

## Introduction

Skin tumors, particularly cutaneous squamous cell carcinoma (cSCC), are among the most common malignancies in humans, and their incidence continues to rise (1). They occur predominantly in sun-exposed areas, such as the face and the head-and-neck region, due to chronic ultraviolet radiation exposure (2). The most frequent malignant skin tumors include basal cell carcinoma, squamous cell carcinoma, and malignant melanoma (3, 4).

Among these, cSCC represents the second most common type and is characterized by a higher metastatic potential compared to basal cell carcinoma. Delayed diagnosis or treatment can therefore result in local tissue destruction and serious complications (5, 6). Whenever feasible, surgical excision is considered the primary and most effective therapeutic option, as it allows for complete tumor removal and significantly reduces the risk of recurrence (7, 8).

However, surgeons have to manage a balancing act between tumor free margins and the preservation or reconstruction of functional and cosmetic structures. Various factors, such as tumor size, guide this therapeutic decision. In cases where excision results in larger defects or occurs in the facial region, complex reconstruction techniques, including flap or full-thickness skin grafting, are often required. Furthermore, synchronous as well as metachronous metastases in the cervical lymph nodes and the parotid gland can lead to necessary surgical resections in terms of a parotidectomy or neck dissection with partially resection of muscular tissue, the jugular veins or cervical nerves e.g. facial nerve or accessory nerve. Literature also reports on metachronous metastases in the parotid gland with potential involvement of the facial nerve and persistent facial nerve paralysis (9, 10).

This retrospective study aims to provide a systematic overview of surgically treated cSCC cases and followed long-term at our clinic. The primary objective is to assess the risk of incomplete (non in sano) resection and recurrence among the included patients. The secondary objective is to evaluate the occurrence of synchronous and metachronous metastases and to determine whether intensive follow-up, including regular ultrasonography of the salivary glands and cervical lymph nodes, could have reduced the extent of resection required, including the need for parotidectomy, which carries the risk of facial nerve paralysis.

## Methods

This retrospective study included patients diagnosed with cSCC in the head and heck who received treatment between January 2019 and December 2024 at the Department of Oto-Rhino-Laryngology, Head and Neck Surgery, Medical Center – University of Freiburg, and the Faculty of Medicine, University of Freiburg.

Comprehensive data were extracted from institutional databases, including both internal and external pathology reports. The evaluated parameters comprised histopathological subtype, depth of invasion, surgical safety margins, and tumor thickness. In addition, photographic documentation, follow-up records, operative reports, discharge summaries, and outpatient visit notes were reviewed. Ultrasound findings and corresponding imaging documentation were examined and cross-referenced to verify consistency across data sources.

Data compilation and documentation were performed using Microsoft Excel, and statistical analyses were conducted with IBM SPSS Statistics software (version 29.0.0.0). All patient data were pseudonymized prior to analysis in accordance with institutional ethical and data protection standards.

The study protocol was reviewed and approved by the Ethics Committee of the Albert-Ludwigs-University of Freiburg (approval number 24-1493-S1-retro). The study is registered in the Freiburg Clinical Trials Registry (No. 006002) and in the German Clinical Trials Register (DRKS No. DRKS00037588).

Patient information was obtained from digital hospital records and included demographic variables, tumor characteristics, surgical details, and histopathological findings, as described in the previous section. Both, clinical (cT) and pathological (pT) tumor stages were assessed according to the TNM classification (TNM Classification, UICC manual, 8^th^ edition), as far as retrospectively available.

Eligibility criteria included a confirmed histopathological diagnosis of cSCC located within the head and neck region. Only patients aged 18 years or older who underwent initial curative-intent surgical treatment at our institution were included. Patients with incomplete medical records, prior surgical treatment were excluded.

Recurrence-free survival (RFS) was assessed using Kaplan–Meier survival analysis, with intergroup differences evaluated by the log-rank test. Associations between histopathological (e.g., subtype, invasion depth, tumor diameter, tumor location) and clinical variables (sex, age) with resection margin status or recurrence were assessed. using the chi-square test. The optimal cut-off value for tumor depth was determined using a receiver operating characteristic (ROC) analysis. A p-value of < 0.05 was considered statistically significant. Multivariable analyses were not performed due to the limited number of events, as this would have resulted in unstable models with an increased risk of overfitting.

R0 was defined as complete excision with a safety margin of at least 3 mm or histopathological confirmation of complete tumor removal with tumor-free resection margins, independent of preoperative tumor size or T-stage. Patients who underwent simple biopsy were classified as R1, as this procedure serves only to establish the tumor entity and does not aim to achieve R0 resection.

## Results

### Age and sex distribution

The mean age at diagnosis for patients with cSCC of the head and neck was 80.78 years (SD = 0.77; 95% CI: 79.25–82.30), with a median age of 82.50 years. The cohort included 153 males (86.9%) and 23 females (13.1%), representing a male-to-female ratio of 6.7:1.

### Tumor localization

Within the cohort, the auricular region was the most frequently affected site (n = 106, 60.2%), comprising the auricle without external auditory canal involvement (n = 79), the auricle with canal involvement (n = 6), the retroauricular region (n = 9), the preauricular region (n = 10), and the infraauricular region (n = 2).

**Figure 1:**
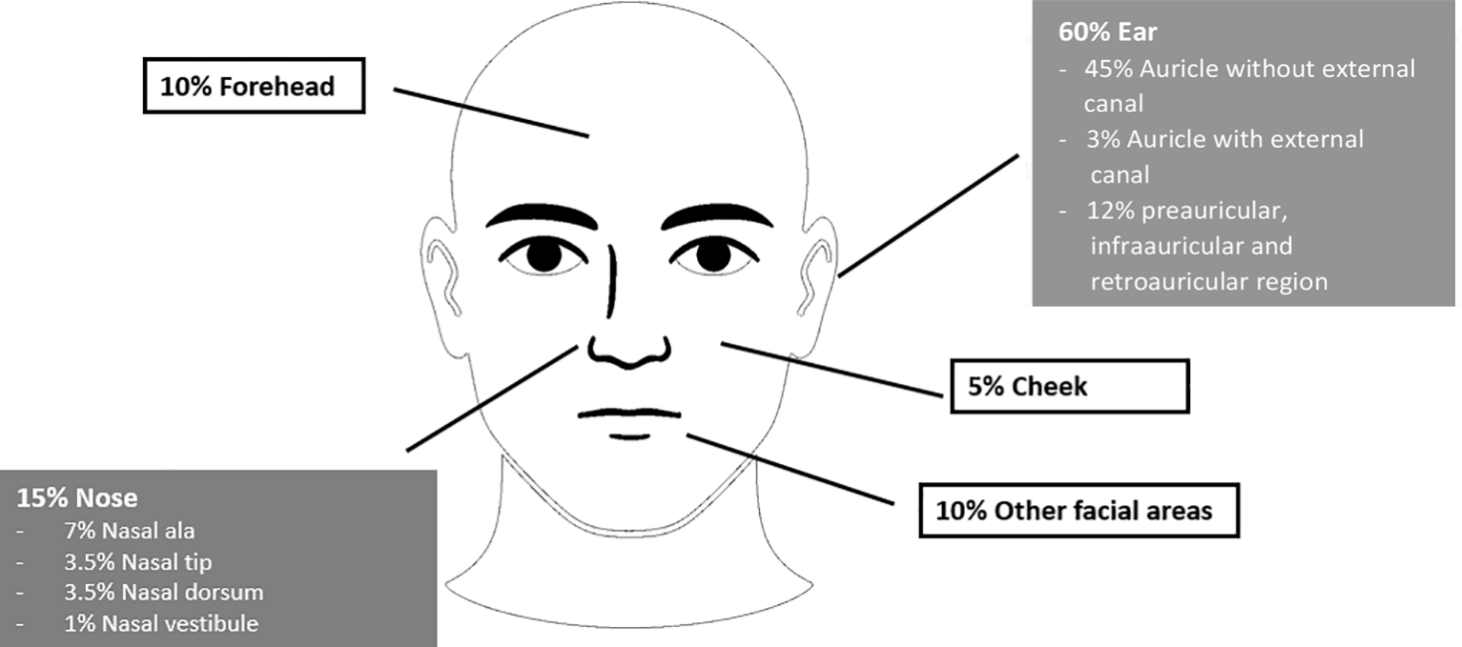
Tumor localization in the head and neck region: ear (60%), nose (15%), forehead (10%), other facial sites (10%) and cheek (5%).

The nasal region accounted for 26 cases (14.8%), including the nasal ala (n = 12), nasal tip (n = 6), nasal dorsum (n = 6), and nasal vestibule (n = 2). The forehead was involved in 18 cases (10.2%), and the cheek in 9 cases (5.1%). Other facial regions accounted for 17 cases (9.7%), involving the eyelid, perioral region (excluding the vermilion), vermilion of the lip, chin, scalp vertex, parietal scalp, and occipital scalp (**Figure 1**).

### Histopathological subtypes

**Figure 2:**
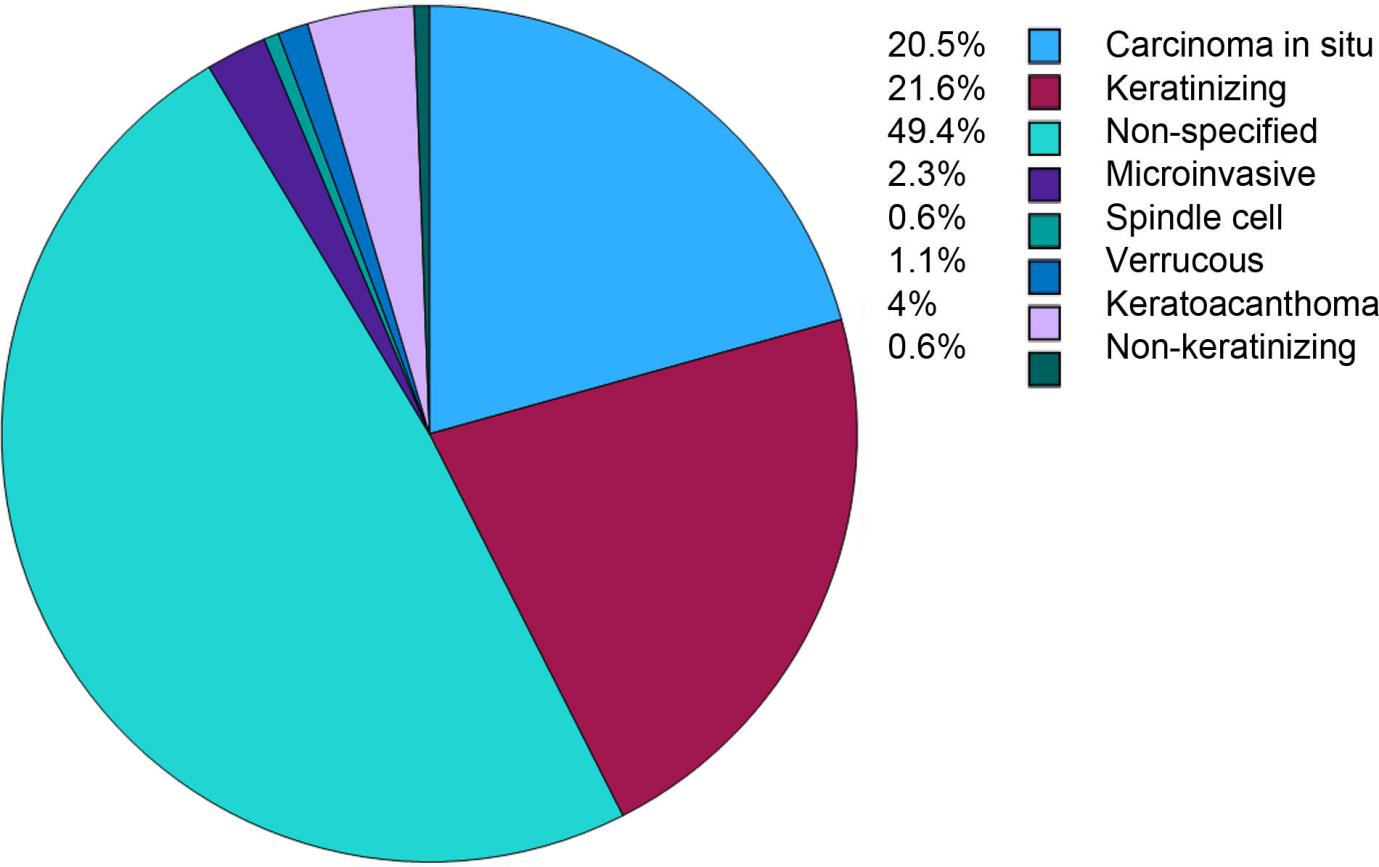
Distribution of histological subtypes of cSCC, with carcinoma *in situ* (20.5%) and keratinizing (21.6%) being most common among specified cases.

Among the 176 patients with head and neck cSCC, carcinoma *in situ* (CIS) was diagnosed in 36 cases (20.5%), keratinizing cSCC in 38 cases (21.6%), and microinvasive cSCC in 4 cases (2.3%). Verrucous carcinoma accounted for 2 cases (1.1%), spindle cell carcinoma for 1 case (0.6%), keratoacanthoma-type cSCC for 7 cases (4.0%), and non-keratinizing cSCC for 1 case (0.6%). In 87 cases (49.4%), the histopathological subtype was not specified (**Figure 2**).

### Tumor staging

Tumor staging according to the pathological T-classification was available for 140 out of 176 cases (79.5%). Among these, most cSCC were classified as pT1 (44.3%), followed by pT3 (15.9%) and pTis (12.5%). Only a small proportion were pT2 (6.3%) or pT4 (1.6%).

### Resection status

**Figure 3:**
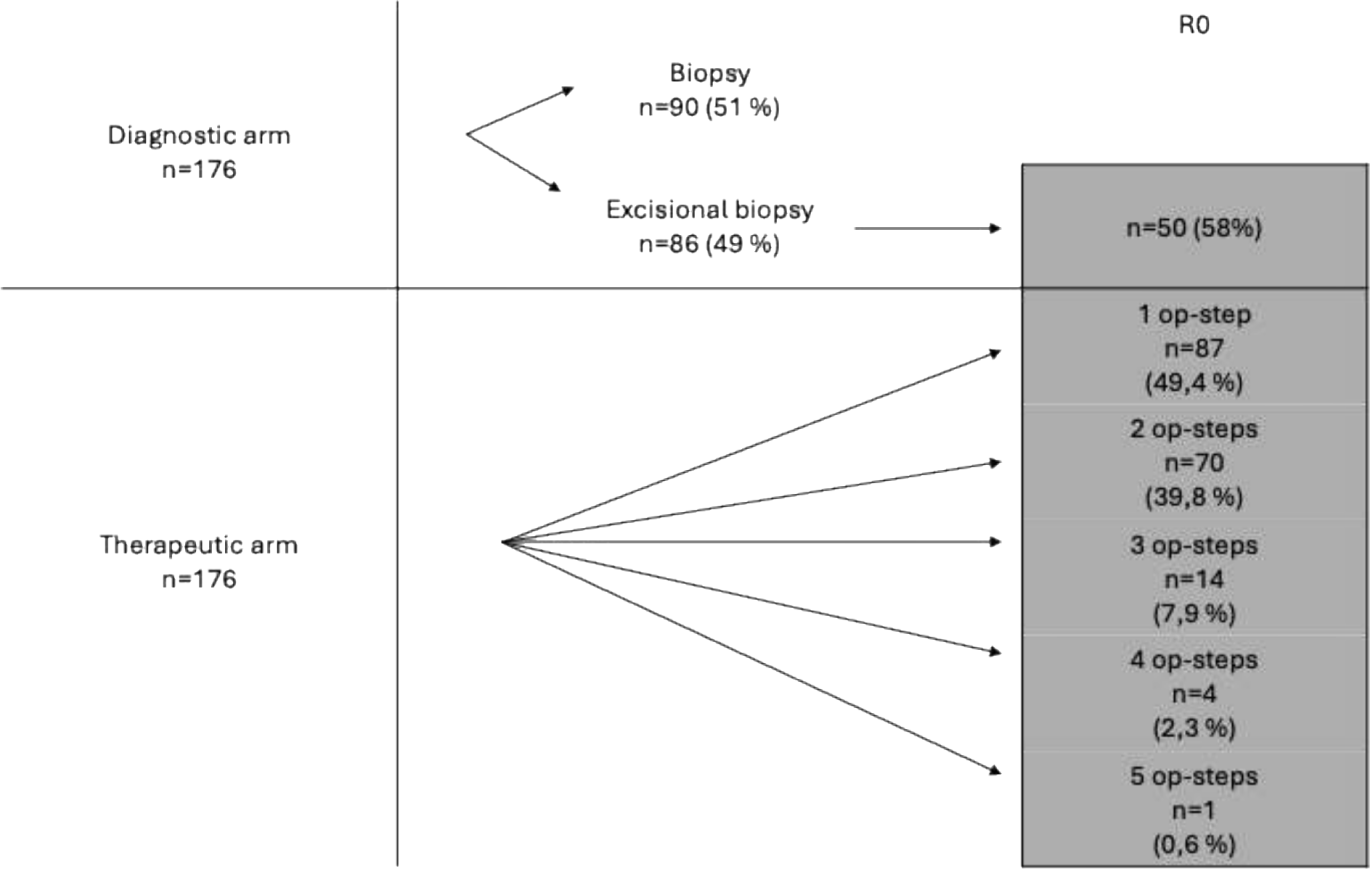
Overview of tissue sampling methods and distribution of the number of surgical stages required to achieve tumor clearance in the therapeutic pathway.

Tissue sampling was performed either by biopsy (n = 90; 51%) or excisional biopsy (n = 86; 49%). Among the excisional biopsies, complete tumor removal (R0 status) was achieved in 50 cases (58%). All patients with non–in sano resections underwent re-excision until R0 status was obtained. In the therapeutic surgical pathway (n = 176), the number of stages required to achieve tumor clearance varied: 87 cases (49.4%) required one stage, 70 (39.8%) required two stages, 14 (7.9%) required three stages, 4 (2.3%) required four stages, and one case (0.6%) required five stages (**Figure 3**).

Regarding surgical margins, tumor size was the only significant factor associated with achieving tumor-free resection margins (R0 status, p < 0.05). No significant association was observed between margin status and histological subtype, tumor stages, sex, age, or depth of invasion (p > 0.05).

### Risk factors for local recurrence

**Figure 4:**
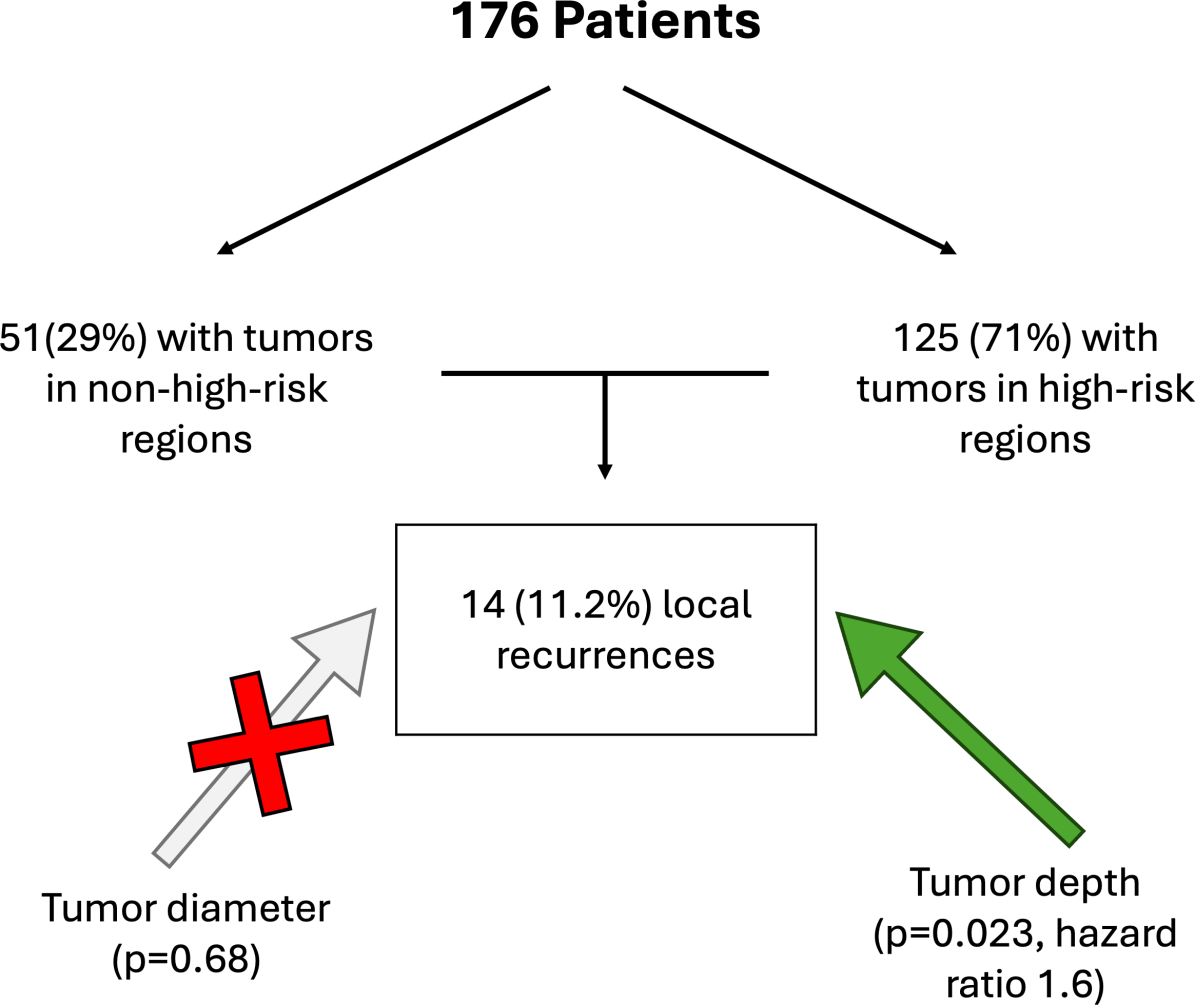
Distribution in relation to risk-dependent localization and local recurrence depending on tumor diameter and tumor depth in patients with cSCC.

**Figure 5:**
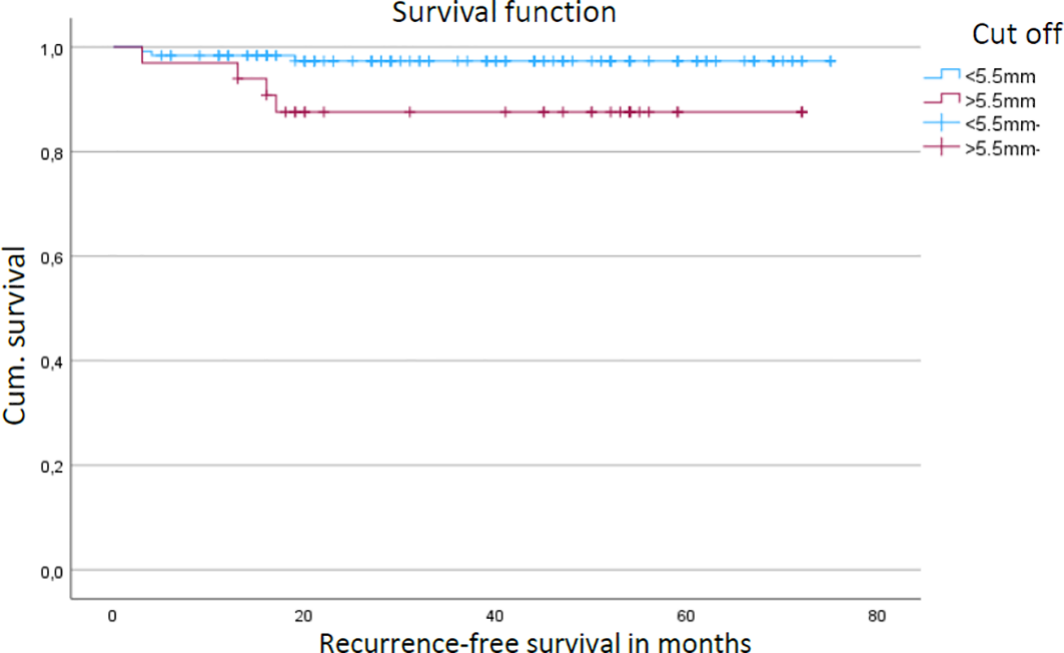
Kaplan–Meier analysis of local recurrence-free survival stratified to tumor depth in patients with cSCC. Blue line – tumor depth < 5.5mm, purple line – tumor depth > 5.5mm.

Most tumors (n = 125, 71%) were located in high-risk anatomical regions (ear and nose), whereas 51 tumors (29%) were located in non–high-risk regions (e.g., cheek or perioral region). During follow-up, 14 patients (11.2%) developed local recurrence, most frequently involving the auricle (n = 8), followed by the nose (n = 3), cheek (n = 1), lip (n = 1), and preauricular region (n = 1). The median follow-up time for the entire cohort was 38.8 months (range 4.6–87.8 months). Among patients who developed local recurrence, the median time to recurrence was 14.05 months (range 3.5–69 months). Tumor diameter did not significantly influence local recurrence-free survival (p = 0.68) (**Figure 4**) as well as tumor stages. In contrast, tumor depth, analyzed as a categorical variable using a cutoff value of 5.5 mm, was significantly associated with local recurrence-free survival (p = 0.023; hazard ratio = 1.6). Tumors exceeding a depth of 5.5 mm showed reduced recurrence-free survival in Kaplan–Meier analysis (**Figure 5**). All patients included in the study ultimately underwent definitive curative-intent surgery, and no patients were lost to follow-up. Tumor depth was ≤ 5 mm in 34.8%, whereas the majority of tumors (65.2%) showed a depth > 5 mm.

### Preoperative ultrasound findings and synchronous and metachronous lymphogenic metastasis

**Figure 6:**
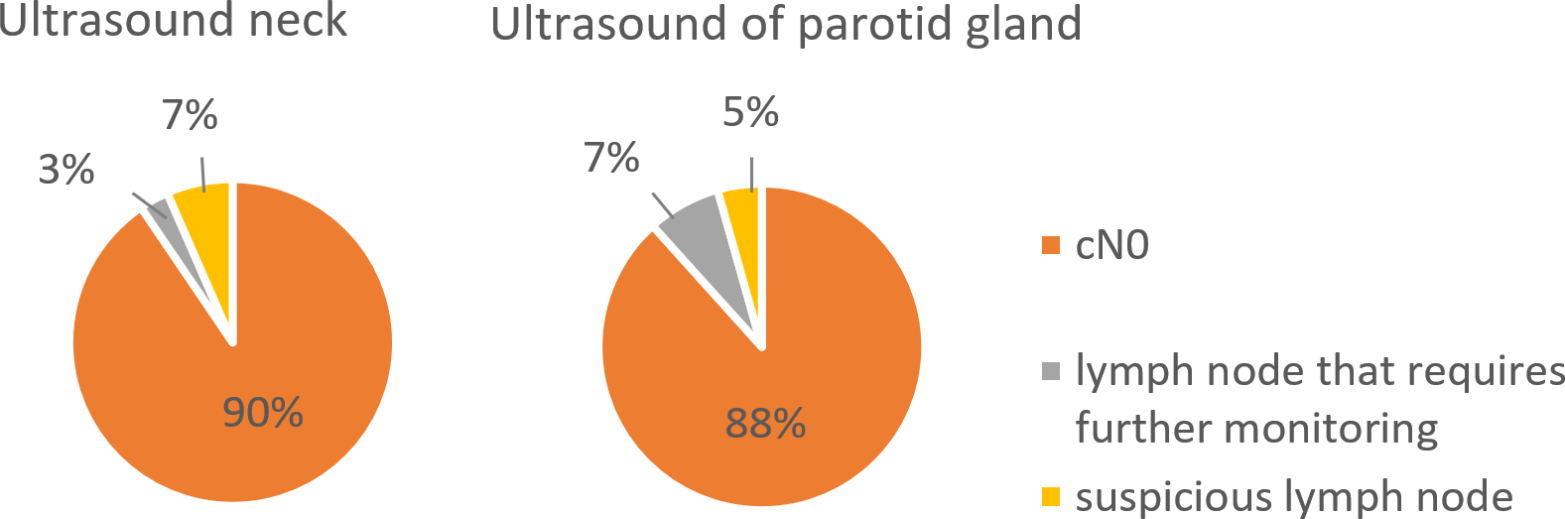
Preoperative ultrasound findings of the neck and parotid gland in patients with cSCC, showing predominantly cN0 status.

Preoperative neck ultrasound revealed no evidence of lymph node metastasis (cN0) in 90% of patients, while 7% had lymph nodes requiring further surveillance and 3% had suspicious lymph nodes. Parotid gland ultrasound showed no suspicious findings (cN0) in 88% of patients, lymph nodes requiring follow-up in 7%, and suspicious lymph nodes in 5% (**Figure 6**). In total, four patients (2.2%) with suspicious lymph nodes underwent neck dissection and/or parotidectomy. In three cases (1.7%), histopathological examination confirmed synchronous lymph node metastases, which were consistently located in cervical lymph nodes but not in the parotid gland.

Five patients (2.8%) developed metachronous lymph node metastases within five years of follow-up and were treated with radical surgery and adjuvant radiotherapy; facial nerve sacrifice was required in two of these cases.

## Discussion

In this cohort of 176 patients with cSCC of the head and neck, the median age at diagnosis was over 80 years. Large population-based and single-center studies report similar median or mean ages in the late 70s to early 80s, with most cases occurring in patients over 70 years (11–13). The marked rise in incidence beyond 75 years and the predominance of head and neck lesions in this age group are attributed to cumulative ultraviolet exposure and other age-related factors (6). The male predominance observed in our cohort is likewise consistent with previous reports, particularly in elderly populations (14, 15).

The predominance of lesions in the auricular and nasal regions in our cohort is also consistent with previous reports, which highlight these sun-exposed and anatomically complex sites as particularly susceptible to ultraviolet radiation–induced carcinogenesis (16). Both areas are classified as high-risk locations for cSCC in current clinical guidelines, warranting more aggressive treatment strategies and closer follow-up to minimize the risk of recurrence and metastasis (6, 17).

Histopathological subtyping in our cohort most frequently identified keratinizing and *in situ* forms, while rare variants such as spindle cell, verrucous, and keratoacanthoma-type cSCC were uncommon. Given that certain subtypes (e.g., spindle cell, acantholytic, adenosquamous, desmoplastic) are linked to more aggressive behavior and poorer prognosis, whereas others (e.g., verrucous, keratoacanthoma-type) tend to have more favorable outcomes, the absence of subtype specification in nearly half of the pathology reports represents a significant gap (18, 19). This finding largely reflects the retrospective nature of the study and the fact that detailed histopathological subtyping is not yet routinely or uniformly reported in cSCC, in contrast to basal cell carcinoma, where subtyping is more standardized (4). The lack of standardized reporting may hinder accurate risk assessment, prognostication, and tailored treatment planning, particularly for tumors in high-risk sites like the auricle and nose. Therefore, detailed subtyping of cSCC is strongly advised to ensure accurate classification and management.

Overall, pT1 and pTis lesions accounted for more than half (56.8%) of all valid cases showing that most cases were diagnosed at an early stage. This is consistent with previous studies reporting that early-stage disease is the most common presentation of cSCC (20, 21). These results suggest that current diagnostic approaches and awareness contribute to early detection before high-risk or advanced tumors develop.

Tumor size was the only significant predictor for achieving R0 resection. Larger cSCCs are more likely to exhibit subclinical extension, reducing the likelihood of complete excision. Tumors ≥2 cm therefore require wider safety margins to ensure complete tumor clearance, which is in line with recent published guidelines and studies (22, 23).

Mohs micrographic surgery represents an alternative surgical approach, particularly recommended for aggressive, high-risk, or recurrent cutaneous squamous cell carcinoma, as it allows for complete margin control while preserving healthy tissue. Several studies have demonstrated lower local recurrence rates for Mohs surgery compared with conventional excision in selected high-risk settings (6, 7, 24). In the present study, Mohs surgery was not performed, as all patients were treated according to institutional standards using conventional surgical excision with histopathological margin assessment.

In our cohort, 11.2% of patients developed local recurrence, markedly lower than recurrence rates reported in elderly patients (median age >80 years) with head and neck cSCC, where approximately 31% of cases recur at both 3 and 5 years, particularly in high-risk sites such as the auricle and nose and in tumors with features such as perineural invasion or poor differentiation (25, 26). Overall, our findings are consistent with previous reports describing local recurrence rates of 11–13% (27–29), and up to 20% in high-risk cohorts (30), typically occurring within the first two postoperative years. The median follow-up time of the overall cohort was 38.8 months, whereas the median time to local recurrence was 14 months. This marked difference indicates that, despite heterogeneous follow-up and shorter observation periods for some patients treated later in the study period, the follow-up duration was sufficient to capture the majority of clinically relevant local recurrences. This is in line with previous reports showing that most local recurrences of cSCC occur within the first two postoperative years (31, 32).

The observed lymphatic metastasis rate of 2.8% likewise falls within the lower range of published data, which report nodal spread in 3–15% of cases (33–35). While this comparatively low metastasis rate may in part reflect the high proportion of early-stage tumors in our cohort, it also may reflect the benefits of structured surveillance with routine sonographic imaging, allowing earlier detection and thereby facilitating timely, less morbid interventions. This underscores the clinical relevance of close follow-up strategies, particularly in patients with established risk factors such as increased tumor depth and advanced stage (pT3).

Tumor depth >5.5 mm emerged as a significant adverse prognostic factor for local recurrence-free survival, whereas tumor diameter showed no impact. This finding supports prior evidence (35, 36) indicating that vertical invasion depth has greater prognostic relevance than horizontal extension. Previous studies and meta-analyses have consistently demonstrated that increased tumor depth is associated with a substantially higher risk of local recurrence, metastasis, and disease-specific mortality, even after adjusting for tumor diameter and other risk factors (36, 37).

Preoperative ultrasound of the neck revealed no evidence of nodal metastasis (cN0) in most patients, with only a small proportion showing lymph nodes requiring further surveillance or suspicious findings. Similarly, sonographic evaluation of the parotid gland identified suspicious nodes in only 5% of cases. A review of the literature revealed that suspicious findings on ultrasound have high sensitivity (91%) but moderate specificity (78%), and a negative predictive value of 99%, meaning that a negative ultrasound is highly reliable for ruling out metastasis, but positive findings require histopathological confirmation (14). More broadly, this approach aligns with recent oncologic studies emphasizing the importance of individualized risk stratification, stage-adapted treatment decisions, and tailored surveillance strategies across different solid tumor entities (38–42).

Limitations of this study include its retrospective design and a relatively short follow-up period of up to six years, which may limit the detection of late recurrences. Follow-up duration was heterogeneous, and the high proportion of early-stage tumors (pT1 or *in situ*) likely contributed to the low rate of lymph node metastases. While structured ultrasound surveillance enables earlier detection of nodal disease, it does not influence the biological incidence of metastasis and should therefore be interpreted with caution. The identified tumor depth cutoff of 5.5 mm was derived from ROC analysis within the present cohort and has not been externally validated. Therefore, this threshold should be interpreted as exploratory and requires confirmation in independent cohorts before clinical implementation. Tumor depth was assessed only in cases with an intended primary complete excision; in cases of incisional biopsy or staged re-excision for deep margin involvement, depth was not reassessed, which may have led to an underestimation of the true depth of invasion and should be considered a limitation when interpreting the recurrence analysis. A further limitation is the incomplete reporting of histological subtypes and key pathological risk factors, such as perineural invasion, tumor grade, and lymphovascular invasion, which could not be analyzed despite their established prognostic relevance in cutaneous squamous cell carcinoma.

However, a major strength of this study lies in the fact that all patients were followed up including regular ultrasound examinations in a dedicated specialist consultation by the surgical team itself. This ensured standardized postoperative evaluation, minimized interobserver variability, and increased the reliability of recurrence assessments.

## Conclusion

Tumor depth is considered an important prognostic factor in cutaneous squamous cell carcinoma and may help identify patients at increased risk for local recurrence. Based on risk stratification, patients who may benefit from closer follow-up, including head and neck ultrasonography, can be identified. A risk-adapted surgical and follow-up strategy may help optimize oncologic outcomes while minimizing overtreatment.

## Data Availability

The raw data supporting the conclusions of this article will be made available by the authors, without undue reservation.
